# Added value of hybrid SPECT with CT imaging for predicting poor therapeutic efficacy of ^89^Sr in patients with bone metastasis

**DOI:** 10.1038/s41598-020-78372-5

**Published:** 2020-12-03

**Authors:** Ben Meng, Jia Song, Lisheng Liu, Longlan Chen, Xiaoliang Chen

**Affiliations:** 1 Chongqing Key Laboratory of Translational Research for Cancer Metastasis and Individualized Treatment, Chongqing University Cancer Hospital, Chongqing, 400030 China; 2 Key Laboratory for Biorheological Science and Technology of Ministry of Education (Chongqing University), Chongqing University Cancer Hospital, Chongqing, China

**Keywords:** Bone metastases, Therapeutics, Quality of life

## Abstract

To utilize single-photon emission computed tomography/computed tomography (SPECT/CT) scanning to investigate the usefulness of nerve root compression (NRC) and radioactive cold zone lesions (RCZLs) for predicting poor therapeutic efficacy of strontium-89 chloride (Sr-89) in patients with bone metastasis. Patients with bone metastatic neoplasms who had undergone baseline bone SPECT/CT scanning before Sr-89 therapy (148 MBq Sr-89 chloride by an intravenous injection for each patient) between July 2011 and July 2018 were included. Bone SPECT/CT images were assessed by two readers independently. Associations between imaging features and therapeutic efficacy were obtained via multivariate logistic regression analysis. Of 231 patients analyzed, 50 (21.6%) had NRC at baseline. Of 31 patients who experienced poor therapeutic efficacy, 29 (93.5%) had NRC. In multivariate logistic regression analysis baseline NRC independently predicted poor therapeutic efficacy. The sensitivity of NRC for predicting poor therapeutic efficacy was 93.5%, specificity was 89.5%, positive predictive value was 58.0%, and negative predictive value was 98.9%. RCZLs were detected in17 patients (7.4%), of whom 14 experienced poor Sr-89 therapeutic efficacy. The sensitivity of the presence of RCZLs for predicting poor therapeutic efficacy was 45.2%, specificity was 98.5%, positive predictive value was 82.4%, and negative predictive value was 92.1%. After adjusting for age, bone metabolism and lesion type, the significant independent predictors of poor Sr-89 therapeutic efficacy were presence of NRC (*p* < 0.001) and RCZL (*p* = 0.001). NRC and RCZL on baseline bone SPECT/CT are reliable independent predictors of poor Sr-89 therapeutic efficacy in patients with bone metastasis. These associations may facilitate the administration of more effective therapeutic interventions.

## Introduction

The incidence of bone metastasis is pretty high in advanced breast, prostate and lung cancer. Above 80% of the patients with symptomatic bone metastatic have tumors originating in breast cancer or prostate cancer^1–3^. The most commonly associated symptoms are severe pain, spinal cord compression, and pathological fractures, of which bone pain from symptomatic skeletal lesions often compromises mobility and the quality of sleep. The associated increased medical costs and reduced survival diminish patient quality of life substantially. Approximately 65% of patients with bone metastases report bone pain^4–8^. To reduce bone pain, improve quality of life, and further control the development of bone metastasis, treatment methods including surgery, external irradiation, chemotherapy, hormone therapy and radionuclide therapy have been utilized. Each treatment method has specific clinical indications and characteristics^9–11^. Surgical intervention was usually required when severe cord compression occurs, the unstable spine needs support or pathologic fractures require stabilization for further therapy^9^. External beam radiation therapy, including local and wide-field radiation, can efficiently reduce pain within 48 h since therapy starts. However, patients with widespread lesions who need higher radiation dose may suffer from significant side effects, particularly myelosuppression^12^. Chemotherapy and hormones are more likely to treat diffuse bone pain. As the first line treatment of bone pain, analgesic medication is conducted according to the World Health Organization guidelines, which recommend a progressive 3-step approach based on pain degree, starting from nonsteroidal anti-inflammatory drugs (aspirin, ibuprofen, naproxen, etc.), to weak opioid (codeine or hydrocodone), and then more potent opioids (morphine, hydromorphone, fentanyl, etc.). However, the side effects of these agents, such as complicating the treatment process, and the increasing long-term cost cannot be ignored^9,13^. On the other hand, specific inhibitors (like bisphosphonates, mithramycin, and calcitonin) are playing an increasing role in alleviating bone pain. But these agents usually require a long period of administration to be effective^14,15^. As for hormones therapy, it appears to be effective only when treating breast or prostate cancer patients. And the recurrence of pain also limits its wide application^16^. Of these, systemically radionuclide therapy with suitable radiopharmaceuticals exhibit unique advantages, including the potential abilities of simultaneously treating multiple metastatic sites, rarely conflicts with other treatments, repetitive treatment and facile administration^17^. Several radiopharmaceuticals have been developed for the treatment of painful bone metastases, such as P-32, Sr-89, Sm-153, and Re-186/188 (Table 1)^12^. Although the radiopharmaceuticals with different physical and biochemical characteristics exhibit various onset and duration of response, researches did not show significant differences in the aspect of bone marrow toxicity and response rate^1^. Even so, long-lived radioisotopes (i.e., Sr-89) exhibit longer duration of response than short-lived isotopes (i.e., Sm-153 and Re-186)^18^.

**Table 1:** Radiopharmaceuticals used to treat bone pain.

Radiopharmaceutical	Half-life (days)	Maximum β-energy (MeV) (mean)	γ-Energy (MeV) (%)	Maximum tissue penetration (mm) (mean)
P-32	14.3	1.71 (0.695)	–	8 (3)
Sr-89	50.5	1.46 (0.58)	0.91 (0.01)	5.5 (2.4)
Sm-153	1.9	0.81 (0.23)	0.103 (28)	2.5 (0.6)
Re-186	3.7	1.07 (0.349)	0.137 (9)	4.5 (1.1)
Re-188	0.7	2.12	–	11 (3)

Biologically, Sr-89 behaves like calcium, localizes in bones and primarily concentrates in areas of high osteoblastic activity. To date Sr-89 has been used extensively in the palliation of pain arising from bone metastases, with the aid of single photon emission computed tomography (SPECT)/computed tomography (CT). The usual therapeutic dose is 148–150 MBq or 1.48–2.22 MBq/Kg. Overall, the response rate is around 76%, range from 60 to 90%, and 5–20% of patients gain complete pain relief. The onset of pain palliation generally occurs within 4–28 days, with a mean duration of pain reduction of 6 months. The most common side effects are hematological toxic effects, with a leukocyte and platelet counts decrease of 11–65% at 4th to 6th week after injection in 12–80% of patients^19^. Sr-89 has been approved by the U.S. Food and Drug Administration for general clinical application, and demonstrated safety and therapeutic efficacy. Sr-89 can significantly reduce the toxicity of radiation, and avoids the technical complications associated with large-field external-beam radiotherapy^20,21^.

The administration of Sr-89 can significantly improve quality of life in patients suffering from skeletal pain. In addition, the fusion of SPECT and CT—a modality involving the integration of functional and structural imaging-has been widely used in patients with bone metastases, to evaluate the condition of patients before intervention treatment^22,23^. Notably however, in our previous clinical practice, we encountered a proportion of patients with bone metastases in whom the therapeutic efficacy of Sr-89 was poor. Therefore, better identification of the relevant predictors based on SPECT/CT is crucial for predicting therapeutic effects and choosing optimal therapeutic interventions. In the present study nerve root compression (NRC) and radioactive cold zone lesions (RCZLs) were investigated as potential SPECT/CT imaging-derived predictors of poor Sr-89 therapeutic efficacy in patients with bone metastasis.

## Patients and methods

### Patients

The Ethics Committee of Chongqing University Cancer Hospital approved this study. Patients aged older than 18 years with bone metastases who had undergone baseline bone SPECT/CT scanning prior to Sr-89 therapy at our hospital from July 2011 to July 2018 were screened for inclusion in the study. Patients who had undergone external irradiation or other bone metastases therapies a month before or three months after the Sr-89 therapy were excluded from the study, as were patients who died within 3 months after the initial bone SPECT/CT. Patients who had multiple myeloma associated with bone metastasis or primary bone neoplasm were also excluded. The basic information such as age, gender, and primary tumor type, other relevant data from SPECT/CT including type and number of bone metabolism lesions, RCZLs, the presence of NRC, and surgery targeting the primary tumor were recorded in detail. Therapeutic effects were assessed via interviews of patients by physicians in conjunction with a standard numeric rating scale (NRS) before Sr-89 therapy and every 2 weeks from the initiation of treatment until 3 months after it. Pain data based on a 0–10 NRS served as the self-reported response index. Poor therapeutic efficacy was defined as the minimum value of NRS (in 3 months) remaining unchanged or increasing compared with that before Sr-89 therapy^24,25^. Sr-89 treatment is usually decided by patients' choice and actual conditions. And all patients were informed with the possibility of ineffectiveness before Sr-89 treatment.

### Pretreatment evaluation

Firstly, all patients underwent a ^99m^Tc-MDP bone SPECT/CT scan. The information of bone SPECT/CT scan and the equipment used is as follows: All images were acquired on a hybrid SPECT/CT equipped with a 16-slice multi-detector CT (GE Discovery NM/CT 670; GE Healthcare, USA). Systemic SPECT imaging was performed 3 h after intravenous injection of ^99m^Tc-MDP (740 MBq), and SPECT/CT fusion scanning was performed at local abnormally distribution region of imaging agents. The data were analyzed by Xeleris Workstation (GE Healthcare).

Then the patient who prepared for Sr-89 treatment needs thorough blood count and serum chemistry testing. If any hematological parameters (white blood cell counts should > 2,000/mm^3^, platelet count should > 75,000/mm^3^, hemoglobin should > 9 g/dl, serum creatinine should < 2.0 mg/dl) not fulfill the criteria, the treatment would be postponed or canceled.

### Protocol treatment

The patients received 148 MBq (4 mCi) of Sr-89 chloride by an intravenous injection followed by a 10 mL saline flush.

### Image analysis

**Figure 1:**
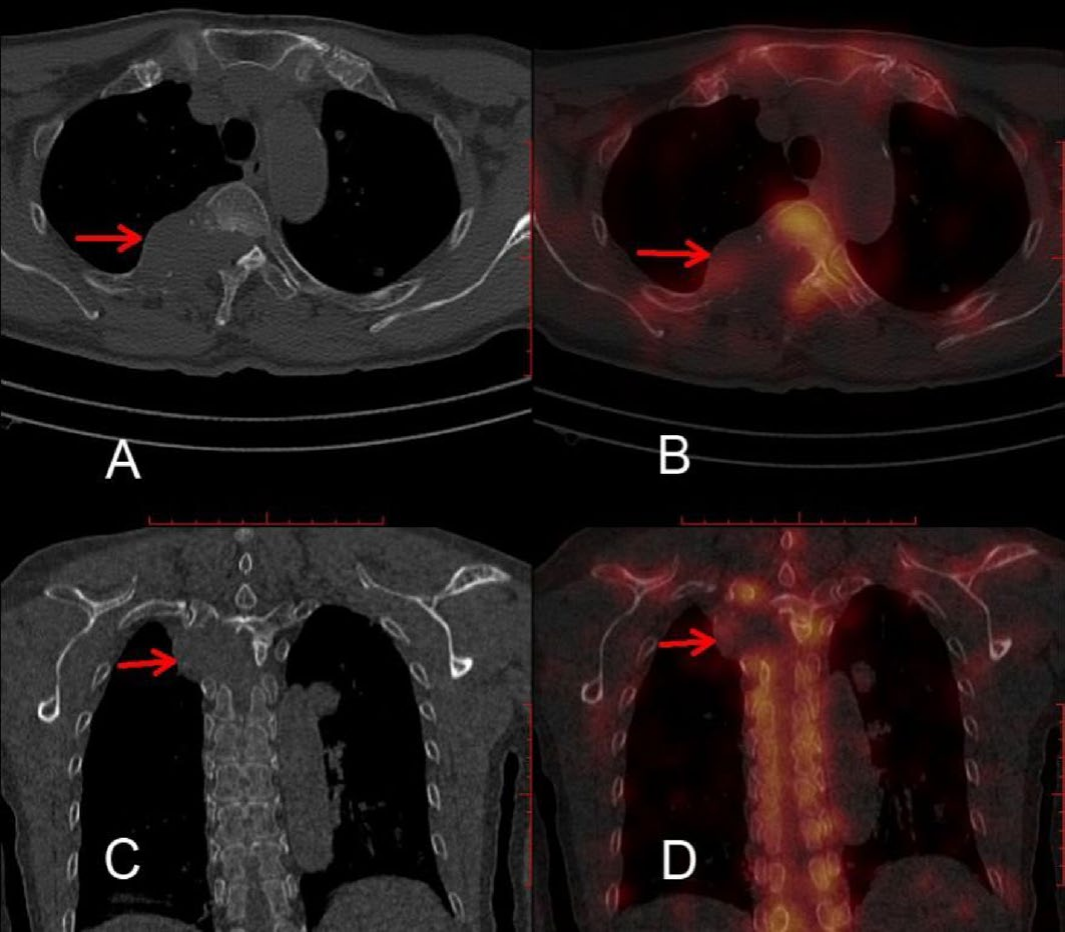
Presence of NRC on bone SPECT/CT scan.

**Figure 2:**
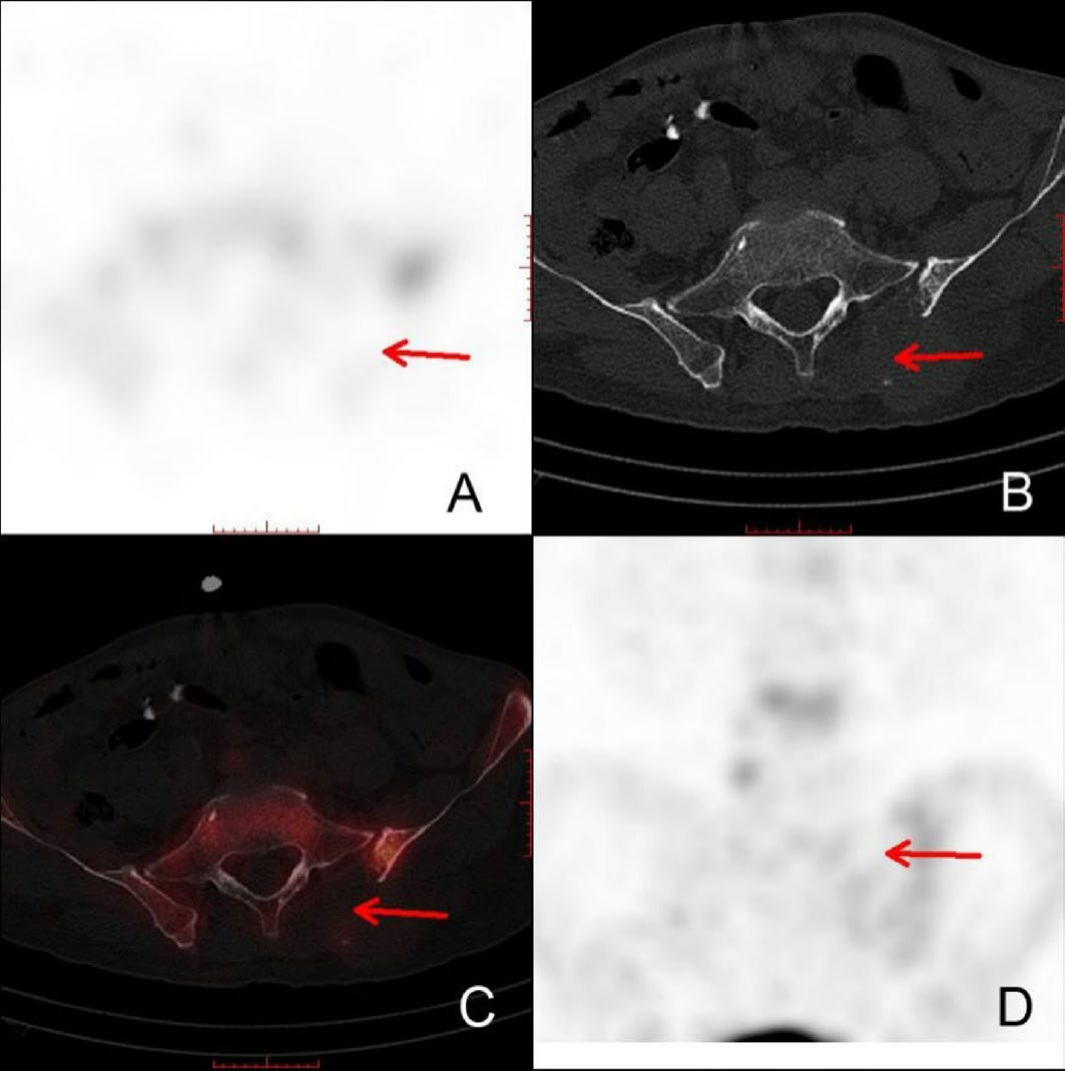
Presence of RCZLs on bone SPECT/CT scan.

The initial bone SPECT/CT images were obtained via the picture archiving and communication system and recorded in Digital Imaging and Communications in Medicine format for review. In this study NRC was identified as a soft tissue density shadow beside a paravertebral mass, as shown in Fig. 1. The clinical profiles of the patients were reviewed by two experienced readers independently to assess the presence of NRC, and discrepancies were resolved via discussion. When two flatly disagreed with each other, the case was settled to consensus by turning to another authoritative reader. RCZLs included the traditionally considered low-concentration of osteolytic hypo concentrated lesions, and also the mixed lesions with concentration at the edge but not at the center (Fig. 2).

### Statistical analysis

All statistical analyses were conducted using an SPSS software package (Version 20.0). Discrete variables were expressed as percentages, and continuous data were expressed as means ± standard deviation (SD) analyzed via *t*-tests. Baseline characteristics including demographic information, clinical data, medical history, and radiological characteristics in the acceptable Sr-89 therapeutic efficacy and poor Sr-89 therapeutic efficacy groups were compared. Multivariate logistic regression analysis was used to determine independent correlations between poor Sr-89 therapeutic efficacy and RCZL and the presence of NRC, and adjusted odds ratios and 95% confidence intervals were calculated. Interobserver reliability with regard to the presence of NRC was determined using κ values. *p* < 0.05 was considered statistically significant.

## Results

### Baseline characteristics

A total of 231 patients were included in the present study, 84 men (36.4%) and 147 women (63.6%), the patients’ mean, minimum and maximum age were 54.9 ± 13.1, 29 and 85 years, respectively. Among the 231 patients the baseline osseous metastases count was less than or equal to 3 lesions in 59 (25.5%), and more than 3 lesions in 172 (74.5%). The bone metastases lesions were all osteolytic in 148 patients (64.1%), osteoblastic in 53 (22.9%), and mixed in 30 (13.0%). There were metastases lesions located only in the skeletal system for 148 patients (64.1%), and in skeletal system together with other systems for 83 patients (35.9%). RCZLs were observed in 17 of the 231 patients (7.4%) in baseline radionuclide bone scans. NRC was observed in 50 of the 231 patients (21.6%) in SPECT/CT scans. The primary tumor was operated on before the baseline radionuclide bone scan in 156 of the 231 patients (67.5%).

### Prevalence and characteristics of NRC and RCZLs

SPECT/CT scanning depicted NRC in 50/231 patients (21.6%) at baseline. NRC was observed in 29 of the 31 patients who exhibited poor Sr-89 therapeutic efficacy (93.5%), whereas it was only observed in 21 of the 200 patients who exhibited acceptable Sr-89 therapeutic efficacy (10.5%). The relevant radiological features of patients with and without SPECT/CT NRC are shown in Table 2. The two readers exhibited excellent agreement with regard to identifying NRC. Other values with respect to the use of NRC for predicting poor Sr-89 therapeutic efficacy were sensitivity 93.5%, specificity 89.5%, positive predictive value 58.0%, and negative predictive value 98.9%. The results of univariate and multivariate logistic regression analysis to evaluate associations between clinical and radiological parameters and Sr-89 therapeutic efficacy are shown in Table 3. Univariate analysis indicated that the type of bone metabolism lesion, RCZLs, and the presence of NRC were significantly associated with poor Sr-89 therapeutic efficacy. In multivariate logistic regression analysis RCZLs (OR 90.91, 95% CI 6.99–1165.11; *p* = 0.001) and the presence of NRC (OR 167.50, 95% CI 21.10–1329.78; *p* < 0.001) were significant independent predictors of poor Sr-89 therapeutic efficacy. Of the 17 patients in whom RCZLs were detected at baseline, 14 (82.4%) exhibited poor Sr-89 therapeutic efficacy. Parameters associated with the capacity of RCZLs to predict poor Sr-89 therapeutic efficacy included sensitivity 45.2%, specificity 98.5%, positive predictive value 82.4%, and negative predictive value 92.1% (Table 2).

**Table 2:** Comparison of baseline demographic, clinical, and radiological characteristics between patients with and without therapeutic efficacy by ^89^Sr.

Variables	Therapeutic efficacy		t/χ^2^	p*
	Ineffective (n = 31)	Effective (n = 200)		
Age	50.4 ± 9.7	55.6 ± 13.4	-2.643	0.011
Gender, male, n (%)	10 (32.3%)	74 (37.0%)	0.261	0.610
Number of lesions, ≤ 3, n (%)	12 (38.7%)	47 (23.5%)	3.265	0.071
**Type of lesion**				
Osteolysis n (%)	27 (87.1%)	121 (60.5%)	9.719	0.008
Ossification n (%)	3 (9.7%)	50 (25.0%)		
Mixed type n (%)	1 (3.2%)	29 (14.5%)		
**Presence of RCZL,** n (%)	17 (54.8%)	197 (98.5%)	68.780	< 0.001
**Presence of NRC,** n (%)	29 (93.5%)	21 (10.5%)	109.148	< 0.001
Presence of extra-osseous transfer, n (%)	8 (25.8%)	75 (37.5%)	1.594	0.207
Surgery aimed at primary tumor, n (%)	22 (71.0%)	134 (67.0%)	0.193	0.661

*p < 0.05 was considered to indicate statistical significance.

**Table 3:** Univariate and multivariate analysis of predictors for the therapeutic efficacy of ^89^Sr.

Variable	Odds Ratio	95% Confidence interval	p*
**Univariate analysis**			
Gender	1.233	0.551–2.761	0.610
Age	0.967	0.936–0.998	0.040
Number of lesions	0.486	0.22–1.075	0.075
Type of lesion (osteolysis)	0.415	0.018–9.648	0.029
Type of lesion (ossification)	0.269	0.078–0.927	0.037
Type of lesion (mixed type)	0.155	0.02–1.184	0.072
Radioactive cold zone lesions	55.556	14.085–206.894	< 0.001
Presence of NRC	123.595	27.508–555.316	< 0.001
Presence of extra-osseous transfer	0.580	0.247–1.362	0.211
Surgery aimed at primary tumor	1.204	0.525–2.76	0.661
**Multivariate analysis**			
Radioactive cold zone lesions	90.909	6.993–1165.112	0.001
Presence of NRC	167.499	21.098–1329.778	< 0.001

*p < 0.05 was considered to indicate statistical significance.

## Discussion

Most patients with bone metastases are accompanied by severe and persistent bone pain. Among various treatment methods using in palliating analgesia, radionuclide internal irradiation treatment exhibited obvious advantages in cases of multiple bone metastases and relatively few adverse reactions in comparison with external radiotherapy, chemotherapy or endocrine therapy.

Sr-89 is one of the most commonly used radionuclides for the treatment of bone metastases, but notably it is not flawless. Skeletal metastases may lead to profound pain if treated inadequately with Sr-89. Thus, identifying reliable predictors of poor Sr-89 therapeutic efficacy is necessary to facilitate the avoidance of side effects, treatment risks, and economic waste. Bone metastases could be assessed via imaging methods before treatment. In the present study bone SPECT/CT was performed to identify the locations of abnormal lesions and lesion characteristics. Nerve root involvement of spinal bone in metastases could be identified in images, and it was found that most patients with nerve root involvement had difficulty achieving good pain relief after Sr-89 treatment. This may be due to bone metastases being affected by nerve roots, usually accompanied by a large soft tissue mass, leading to the tumor at the margin of broken bones lack of sufficient amount of radiopharmaceutical irradiation. Thus, in such situations the pain caused by nerve root involvement may not be relieved, and in at least some cases it may not be possible to completely relieve this type of bone metastases-derived pain via internal Sr-89 irradiation. However, in some cases, the pain caused by local effects of bone remodeling and the inflammatory reaction could be partially alleviated by Sr-89. Thus, the overall effect depends on the source of the pain. In addition, the reason for the association between RCZLs and poor Sr-89 therapeutic effect is likely to relate to the lower radioactivity concentration of bone depicted via scanning, reflecting no osteogenic activity (pure lytic bone lesion) or extremely chronic osteogenic activity. In cases of lower-level osteogenesis, it is difficult to aggregate enough Sr-89, and this results in ineffective treatment. However, as shown in Table 2, there are 60.5% of patients with effective treatment have osteolytic lesions. Actually, patients with small and dispersive osteolytic lesions usually received effective treatment. In these cases, Sr-89 gathered in the bone tissues around the small lesions are enough to play a therapeutic role. And the size range of osteolytic lesions for effective treatment may be our next study. In our clinical practice, we will inform patients with all possible results before treatment, including ineffectiveness. Sr-89 treatment is usually decided by patients' choice and actual conditions. For patients with severe bone pain desire to be relieved and choose to be treated with Sr-89, we didn't follow the guidelines exactly for humanistic care.

Bone metastases are usually classified as osteoblasts, osteolytic, and “mixed”. In univariate analysis, three types of bone metastasis showed a statistically significant difference between the effective group and the ineffective group. In multivariate analysis, however, bone metastases type was not an independent predictor of Sr-89 therapeutic efficacy. Notably, it is highly likely that in numerous cases multiple factors affected the efficacy of Sr-89 therapy simultaneously.

The advantages of the present study include its relatively large sample size and the reliability of the clinical research methods utilized. Notably however, the study had several limitations. It was conducted at a single institution, and thus further studies in other institution may be required. The patients in the study had a variety of primary tumors, and this undoubtedly resulted in numerous clinical differences in various contexts.

### Ethical standard

In this study, written informed consent for treatment was obtained from all patients before the initiation of treatment (all the subjects were over 18 years old). The Ethics Committee of Chongqing University Cancer Hospital approved this study. All methods were carried out in accordance with relevant guidelines and regulations. And all patients were informed with the possibility of ineffectiveness before Sr-89 treatment. Sr-89 treatment is decided by patients' choice and actual conditions.

## Conclusions

NRC and RCZLs detected via bone SPECT/CT scanning are reliable independent predictors of poor Sr-89 therapeutic efficacy in patients with bone metastases. This knowledge may facilitate more effective therapeutic interventions and the avoidance of unnecessary economic burden and side effects in the future.
